# Rootstocks with Different Vigor Influenced Scion–Water Relations and Stress Responses in Ambrosia^TM^ Apple Trees (*Malus Domestica* var. Ambrosia)

**DOI:** 10.3390/plants10040614

**Published:** 2021-03-24

**Authors:** Hao Xu, Danielle Ediger

**Affiliations:** Summerland Research and Development Centre, Agriculture and Agri-Food Canada, Summerland, BC V0H 1Z0, Canada; danielle.ediger@canada.ca

**Keywords:** drought resistance, dwarfing rootstock, heat stress, leaf gas exchanges, stomatal characteristics, water use strategy

## Abstract

In recent years, awareness has been raised around the benefits of diversifying rootstocks, in order to enhance tree health and sustain apple fruit production under the influence of climate change. However, performances of many rootstocks under stresses remain unclear. This study aimed to set the first step towards a much-needed comprehensive evaluation on water relationships and stress responses of scion–rootstock systems for the popular apple cultivar Ambrosia^TM^ (*Malus domestica* var. Ambrosia), because its production and horticultural knowledge had been largely limited to the Malling 9 rootstock (M.9). Five rootstocks were evaluated in a greenhouse water deficit experiment and at the onset of heat stress in a field trial in Summerland, British Columbia, Canada. Multiple stress indicators of leaves and fruits were analyzed to elucidate water use strategies and drought resistance mechanisms. The rootstocks led to differences in scion vigor, and stomatal and photosynthetic characteristics. The largest semi-dwarfing Geneva 202 (G.202) demonstrated more water use and higher stress susceptibility. Large dwarfing Geneva 935 (G.935) and Malling 26 (M.26) showed more stringent stomatal control and reduced water use under stresses, typical of a drought-avoidance strategy. The smallest large dwarfing M.9NIC29^®^ and the small dwarfing Budagovsky 9 (B.9) led to smaller and denser stomata. B.9 demonstrated the most stable water status and drought tolerance. The study suggested that scion stress responses were influenced by rootstock vigor and tree water use strategies. It implied the necessity of vigor-specific irrigation management for alleviating stresses and achieving production goals of different rootstocks.

## 1. Introduction

In apple horticulture, the use of rootstock is an effective approach to manage tree growth and yield, to improve resource use efficiency, and to confer scion with desirable traits of resilience against a variety of abiotic and biotic stressors [1]. Limited water transporting capacity in semi-dwarfing and dwarfing rootstocks are often associated with lower root vigor, lower root partitioning of dry matter, smaller numbers of coarse roots and fine roots [2], smaller vessels and lower root hydraulic conductance [3], and thicker root bark [4]. Water transport in the scion–rootstock system may be further restricted by xylem dysfunction and increased resistivity of the graft union [5]. Such limitations constrain scion hydraulics and tree growth, by the well-known mechanism of altering the root-to-shoot hydraulic signals. Other mechanisms, such as interfered systemic chemical communications via endogenous growth regulators, nutrients, RNAs and proteins, are less understood [6,7,8]. Abundant studies have shown that different rootstocks can lead to variations in scion–water relationships, stomatal development and regulation, and xylem hydraulic properties in a number of apple cultivars [3,9,10,11,12,13]. Although rootstocks with different vigor and water transport capacity are assumed to alter the water demand, water use strategies and drought resistance of the scion, it remains a debate whether the performance of smaller rootstocks with lower water demand would necessarily exceed that of larger rootstocks under water-limited conditions, and whether the scions with different growing habits would inversely impact the performance of specific rootstocks. 

Ambrosia^TM^ apple (*Malus domestica* var. Ambrosia) is an economically significant cultivar to the apple industries in Canada, the United States, Chile, and New Zealand. The fruit is well accepted in the international markets, attributed to its supreme texture, unique sweet sub-acid flavor, pleasant bicolor appearance in pink-red and creamy yellow, and good post-storage quality [14]. Its scion is characteristic of moderate vigor, upright growth habit and fruit bearing spurs. Much of its production zone in the northwest coast regions of North America is semi-arid, similar to the Okanagan-Similkameen Valley of British Columbia, where the production is heavily dependent on irrigation. The prevailing high-density plantings rely on the dwarfing rootstocks which have an inherent hydraulic restriction. Water stress resulting from inadequate irrigation under high evapotranspiration can further hinder carbohydrate assimilation and heat dissipation, which causes the trees to become more susceptible to secondary stresses. Under the influence of climate change, the frequency and intensity of drought and heat stress events increase, posing a continuing challenge to tree health, fruit yield, and quality in Ambrosia^TM^ apple production. Such challenges can be mitigated by selecting the appropriate rootstocks. In the last three decades, the majority of Ambrosia^TM^ apple production and a number of horticultural studies have been based on Malling 9 rootstock [15,16]. In recent years, there has been a slow increase in the use of other rootstocks such as Malling 26 (M.26), Budagovsky 9 (B.9), and innovative Geneva rootstocks that possess resistance to orchard biotic stressors such as fire blight, phytophthora, and aphids [17]. A systematic evaluation on a variety of rootstocks is much needed, to facilitate the selection of the appropriate rootstocks that can confer the scion with better water status and more resilience to environmental stresses, and with sustained yield and fruit quality. 

According to previous studies on common apple cultivars [18,19], five rootstocks representing three vigor levels were assessed in this study. B.9 (M.8 × Red Standard) is typically small dwarfing with good hardiness. Malling 9, M.26 and Geneva 935 (G.935) are usually classified as large dwarfing rootstocks. Malling 9NIC29^®^ (M.9 in the following text) tends to have vigor similar to Malling 9 Pajam2, being one of the most vigorous Malling 9 selections [18,19]. M.26 (Malling 16 × M.9) is one of the more vigorous dwarfing rootstocks, and is sometimes considered as semi-dwarfing, with yield efficiency similar to M.9 [18,19]. G.935 (Ottawa 3 × Robusta 5) has tree size slightly larger than M.26, and very high cumulated yield efficiency [19]. Geneva 202 (G.202; Malling 27 × Robusta 5) is the only semi-dwarfing in the five rootstocks. In a study on Honeycrisp (*M. domestica* var. Honeycrisp), it was about 50% larger than M.9, and had lower yield efficiency but higher cumulative yield [18]. 

To elucidate how the rootstocks with different vigor would alter the water relationships and stress responses of Ambrosia^TM^ scion, scion performances on these five rootstocks were evaluated in a water deficit experiment in the greenhouse, and at the onset of heat stress in a field trial at Summerland Research and Development Centre, Agriculture and Agri-Food Canada (Summerland, British Columbia, Canada). Multiple stress indicators associated with photosynthesis, tree–water relationships and fruit quality, i.e., leaf gas exchange rates, stomatal conductance, maximum quantum efficiency of CO_2_ assimilation, photosynthetic electron transport rate, variable fluorescence: maximum fluorescence ratio, leaf chlorophyll concentration, defoliation, surface temperature of leaves and fruits, fruit water potential, fruit weight, fruit dry matter, and soluble solids content, were analyzed to investigate water use strategies and possible mechanisms underlying drought susceptibility and resistance (i.e., avoidance and tolerance). The responsiveness and causal relationship of the indicators were evaluated to rank their sensitivity to water status variations rendered by rootstocks and stresses. The study highlighted the sensitive stress indicators, improved the understanding of water use strategies and stress responses of the scion–rootstock systems with different vigor levels, and pointed out the advantages and limitations of each rootstock.

## 2. Results

### 2.1. Greenhouse Experiment: Responses of Different Rootstocks to Water Deficit 

#### 2.1.1. Soil Water Depletion and Leaf Gas Exchanges

**Figure 1:**
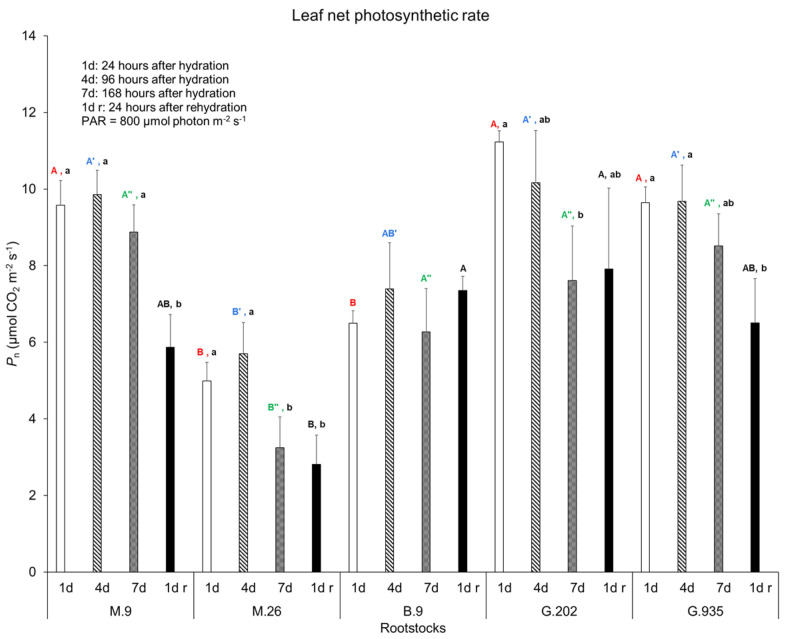
Leaf net photosynthetic rate of Ambrosia^TM^ scion on five rootstocks in the greenhouse water deficit experiment. Bars and error bars are the mean and standard error (*n* = 6). Different uppercase letters in the same color indicate significant differences amongst rootstocks on each day (red, blue, and green for Day 1, Day 4 and Day 7 after irrigation, respectively; black for one day after rehydration) (*P* ≤ 0.05, ANOVA, Fisher LSD pairwise comparisons). Different lowercase letters in each rootstock indicate significant day-to-day variations (*P* ≤ 0.05, ANOVA, Fisher LSD Pairwise Comparisons).

Soil volumetric water content (*VWC*) declined from Day 1 to Day 7 after irrigation (Table 1). Water depleted significantly more in M.9 (−0.31 m^3^/m^3^) and G.935 (−0.3 m^3^/m^3^), than in M.26 (−0.14 m^3^/m^3^) (Table 1). The leaf gas exchanges were assessed during the soil water depletion. On Day 1, M.9, G.202 and G.935 demonstrated higher net photosynthetic rates (*P*_n_) than M.26 and B.9 (Figure 1). On Day 4 and Day 7, M.26 showed lower *P*_n_ than the other rootstocks. From Day 1 to Day 4, none of the rootstocks showed significant decreases in *P*_n_. From Day 1 to Day 7, the *P*_n_ decreased in M.26 and G.202, but it did not change significantly in others. The maximum quantum efficiency of CO_2_ assimilation (*ΦCO*_2_) also declined the most in G.202 (Δ*ΦCO*_2_ at −5.37 mmol CO_2_ mol^−1^ absorbed quanta), followed by M.26 (−2.59 mmol CO_2_ mol^−1^ absorbed quanta), while the extent of decrease in other rootstocks was significantly less (Table 1). Rehydration led to a further decrease in *P*_n_ in M.9 and G.935, but not in M.26, B.9 or G.202. Overall, during soil water depletion, M.26 demonstrated the lowest *P*_n_, whereas B.9 showed the most stable *P*_n_ (Figure 1) and the least decrease in *ΦCO*_2_ (Table 1).

**Figure 2:**
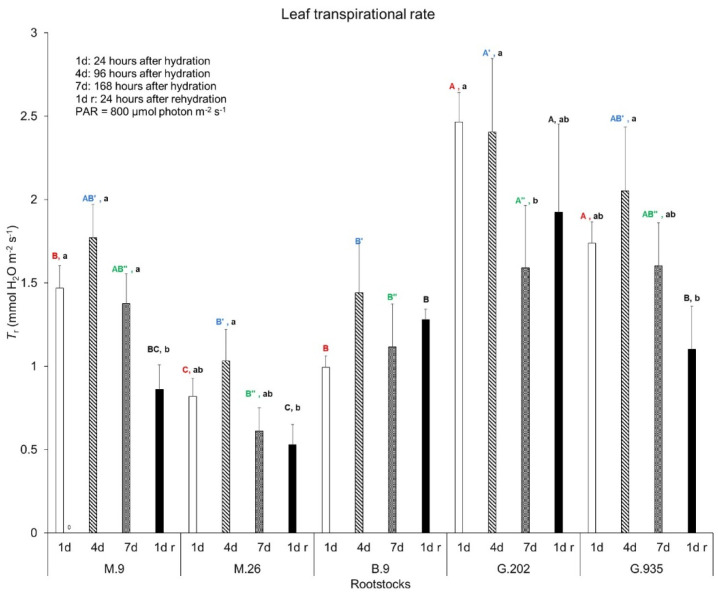
Leaf transpirational rate of Ambrosia^TM^ scion on five rootstocks in the greenhouse water deficit experiment. Bars and error bars are the mean and standard error (*n* = 6). Different uppercase letters in the same color indicate significant difference amongst rootstocks on each day (red, blue, and green for Day 1, Day 4 and Day 7 after irrigation, respectively; black for one day after rehydration) (*P* ≤ 0.05, ANOVA, Fisher LSD pairwise comparisons). Different lowercase letters in each rootstock indicate significant day-to-day variations (*P* ≤ 0.05, ANOVA, Fisher LSD pairwise comparisons).

Transpiration rate (*T*_r_) changed in a pattern similar to *P*_n_. On Day 1, G.202 and G.935 demonstrated a higher *T*_r_ than M.9 and B.9, and M.26 had the lowest *T*_r_ (Figure 2). From Day 1 to Day 4, *T*_r_ slightly increased in all the rootstocks, however the change was not significant. From Day 1 to Day 7, *T*_r_ declined in G.202, but did not show significant change in others (Figure 2). Stomatal conductance (*g*_s_) declined the most in G.202, followed by M.26, G. 935, and M.9. On the contrary, Δ*g*_s_ suggested an increase in B.9 despite its statistical insignificance (Table 1). Rehydration led to a further decrease in *T*_r_ in M.9; however, it did not have any significant impact on others. During the dehydration–rehydration cycle, *T*_r_ in M.9, M.26 and G.935 significantly deceased from Day 4 to one day after rehydration. Overall, M.26 demonstrated the lowest *T*_r_, suggesting the least water use, which was consistent with its least Δ*VWC* in soil (Table 1). B.9 showed a relatively low but the most stable *T*_r_ during soil water depletion. By Day 7, instantaneous water use efficiency (*WUE*_i_) increased the most in G.202, followed by G.935 and M.9 (Table 1). *WUE*_i_ declined more in M.26 than in B.9. 

#### 2.1.2. Stomatal Characteristics 

**Figure 3:**
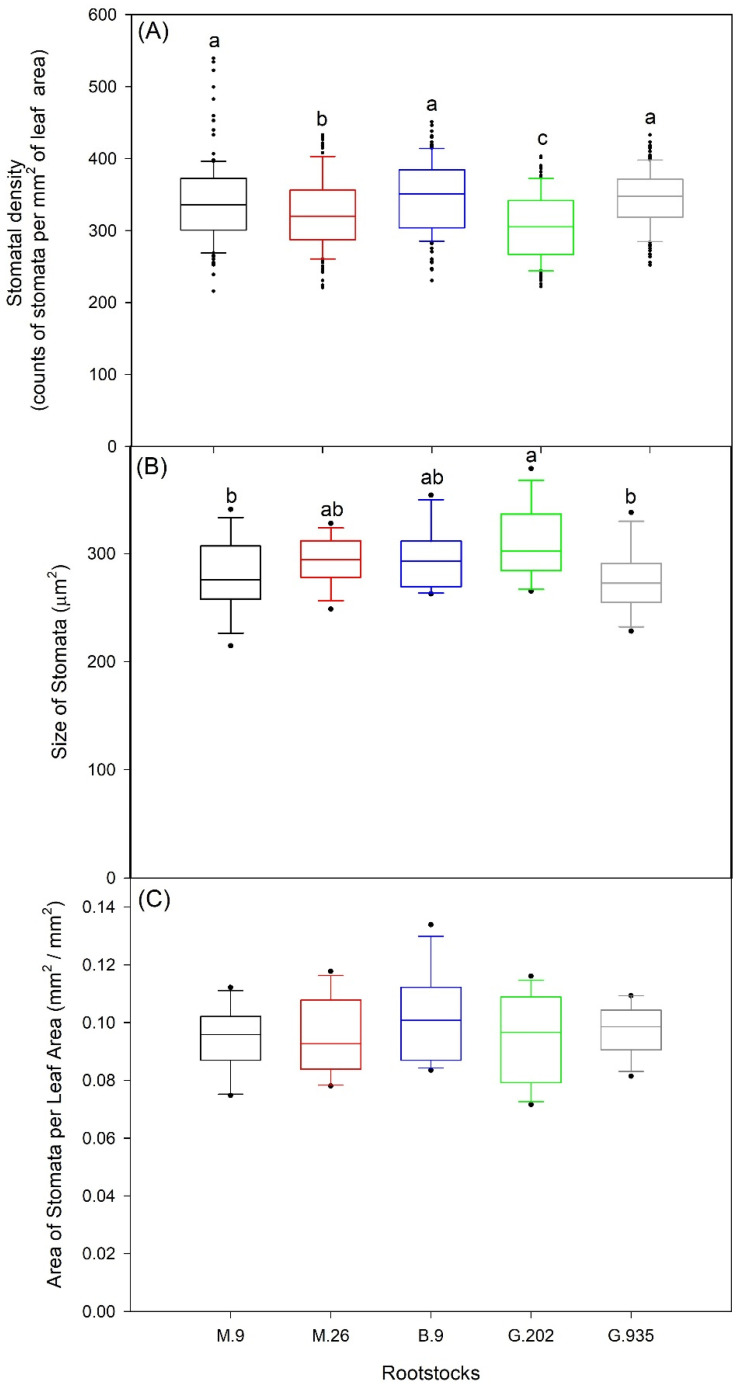
Stomatal density (**A**), average size of stomata (**B**), and stomatal area per leaf area of apple leaves (**C**) of Ambrosia^TM^ scion on five rootstocks in the greenhouse water deficit experiment. Different letters in each sub-panel stand for significant differences amongst rootstocks (*P* ≤ 0.05, ANOVA, Fisher LSD pairwise comparisons) (*n* = 6, each replication consists of 20 imprints, i.e., 5 from each of 4 leaves). The absence of letters means that there were no significant differences.

The stomatal density and size of Ambrosia^TM^ leaves showed variations amongst rootstocks (Figure 3). Stomata of higher density (Figure 3A) and smaller size (Figure 3B) were found in M.9 (341 counts per mm^2^ of leaf area, 280.1 µm^2^ in size on average) and G.935 (345 counts per mm^2^, 275.6 µm^2^). In G.202, the stomata had the lowest density (306 counts per mm^2^) and the largest size (308.3 µm^2^). Compared to M.26 (323 counts per mm^2^, 293.3 µm^2^), B.9 had similar medium-sized stomata (295.9 µm^2^), but at a significantly higher density (347 counts per mm^2^), which was approximate to M.9 and G.935. Area of stomata per leaf area was not significantly different amongst rootstocks, however the median of this parameter was the highest in B.9 (0.101 mm^2^/mm^2^) and the lowest in M.26 (0.093 mm^2^/mm^2^) (Figure 3C). 

#### 2.1.3. Leaf Defoliation, Chlorosis and Scion Trunk Growth

**Figure 4:**
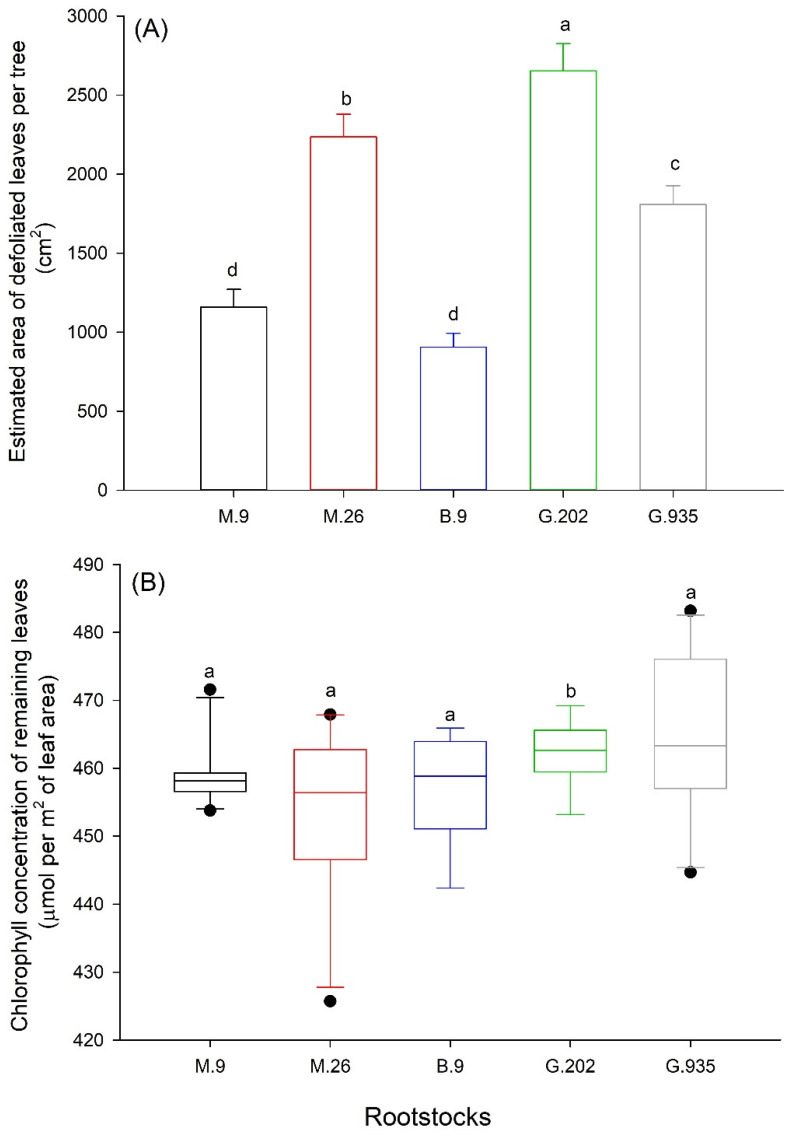
Leaf area of defoliated leaves (**A**) and the chlorophyll concentration of remaining leaves (**B**) of Ambrosia^TM^ apples on five rootstocks by the end of greenhouse water deficit experiment. Different letters in each sub-panel stand for significant difference amongst rootstocks (*P* ≤ 0.05, ANOVA, Fisher LSD pairwise comparisons; *n* = 10).

Soil water depletion caused defoliation. The intensity of defoliation varied amongst rootstocks. The most severe defoliation occurred in G.202, followed by M.26 and G.935; M.9 and B.9 defoliated the least (Figure 4A). G.202 demonstrated higher leaf chlorophyll concentration in the remaining leaves, whereas M.26 showed larger tree-to-tree variation (Figure 4B). 

**Figure 5:**
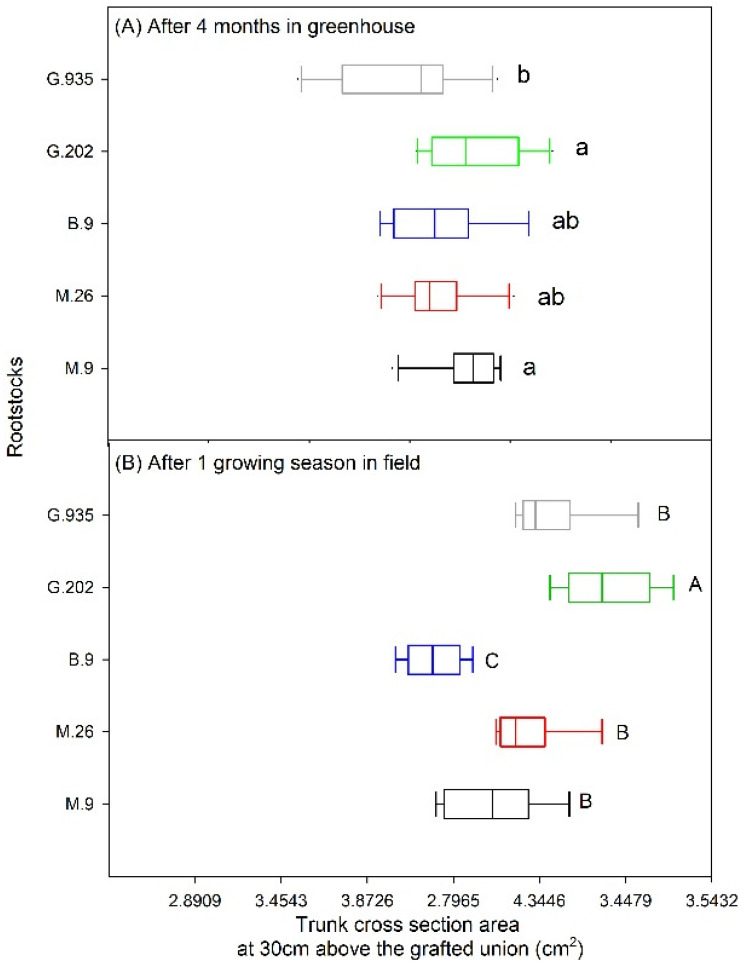
Trunk cross-sectional area of Ambrosia^TM^ scion on five rootstocks after four months in the greenhouse (**A**), and after a growing season in the field (**B**). Different letters in each sub-panel stand for significant differences amongst rootstocks (*P* ≤ 0.05, ANOVA, Fisher LSD pairwise comparisons; (**A**): *n* = 10; (**B**): *n* = 6, each replication consists of 3 trees).

After growing in the greenhouse for four months during October 2018–February 2019, G.202 had the largest scion trunk cross-sectional area (*TCSA*), despite no significant difference from M.9, M.26 or B.9. The *TCSA* in G.935 was significantly smaller than the other rootstocks (Figure 5A).

### 2.2. Field Trial: Rootstock Performances in the Second-Year Planting and Under Heat Stress

#### 2.2.1. Scion Trunk Growth, Yield and Fruit Quality

By the end of the growing season in the field in 2020, G.202 showed the largest *TCSA* and the highest scion vigor, followed by G.935, M.26 and M.9 which demonstrated similar scion vigor. The *TCSA* in B.9 was significantly smaller than others (Figure 5B). Except for B.9, the rootstocks had larger *TCSA* after the growing season in the field than after four-months growth in the greenhouse (Figure 5A,B). Yield efficiency was the highest in B.9, followed by M.9, M.26 and G.935, and was the lowest in G.202 (Table 2). Leaf chlorophyll concentration was also lower in G.202 than in others (Table 2). There was no symptomatic defoliation in any rootstock. 

**Figure 6:**
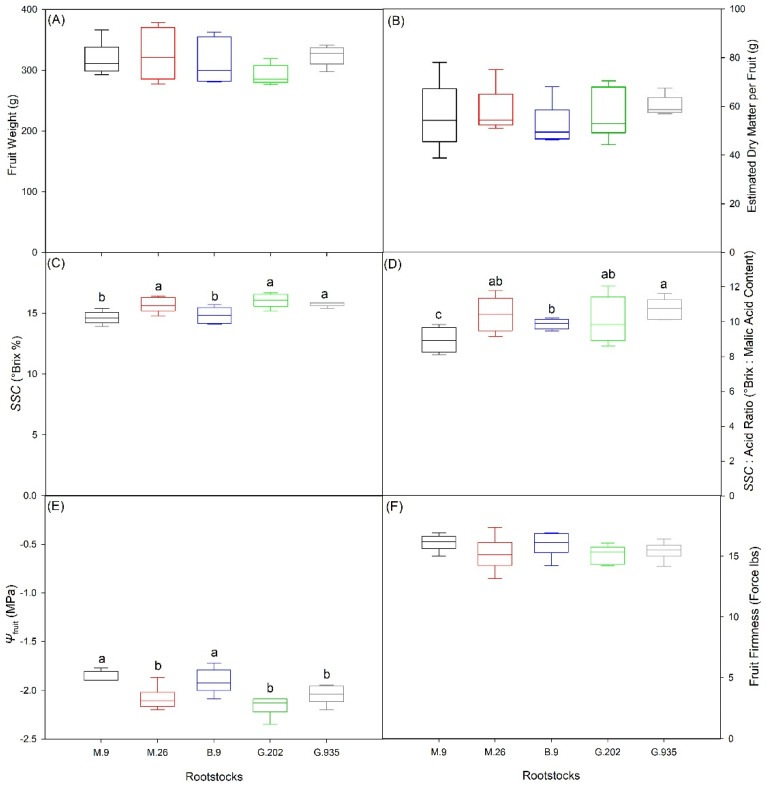
Fruit weight (**A**), dry matter weight (**B**), soluble solid contents *SSC* (**C**), *SSC*: acidity ratio (**D**), water potential of fruit hypanthium *Ψ*_fruit_ (**E**), and fruit firmness (**F**), of Ambrosia^TM^ apples on five rootstocks in the second-year planting. Different letters in each sub-panel stand for significant differences amongst rootstocks (*P* ≤ 0.05, ANOVA, Fisher LSD pairwise comparisons), whereas the absence of letters stands for no significant difference. In (**A**,**B**): *n* = 6, and each replication consisted of 5 fruits; in (**C**,**D**): *n* = 6, and each replication consisted of the juice of 6 fruits; in (**E**,**F**): *n* = 6, and each replication consisted of 4 fruits.

Rootstocks did not lead to significant differences in fruit weight (Figure 6A) or dry matter weight (Figure 6B) (*P* ≤ 0.05). However, fruit weight in G.202 was marginally lower than in G.935 and M.26 (Figure 6A); G.935 had marginally higher fruit dry matter weight with more fruit-to-fruit consistency (Figure 6B). After one month of air storage at 4 °C, the fruits of M.26, G.202 and G.935 had higher soluble solid contents (*SSC*, °Brix %) than M.9 and B.9 (Figure 6C). Sugar: acidity ratio was the highest in G.935, followed by M.26, G.202 and B.9, and was the lowest in M.9 (Figure 6D). Tissue water potential of fruit hypanthium (*Ψ*_fruit_) was higher in M.9 and B.9 than in others (Figure 6E). Fruit firmness was also higher in M.9 and B.9, followed by G.202 and G.935, and was the lowest in M.26 (Figure 6F). Fruit mineral nutrients varied amongst rootstocks, except zinc (Table 2). Fruits of G.935 had higher nitrogen, phosphorous, and boron, but lower potassium, magnesium, and calcium, whereas contrasting compositions were found in the fruits of B.9 (Table 2).

#### 2.2.2. Water Relationships in Response to the Onset of Heat Stress

**Figure 7:**
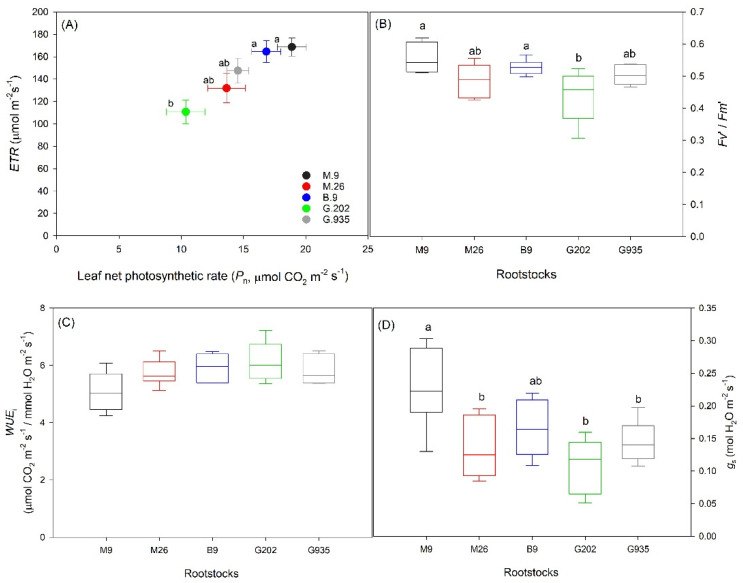
Leaf net photosynthetic rate *P*_n_ and electron transport rate (*ETR*) (**A**), chlorophyll fluorescence *Fv*’/*Fm*’ (**B**), instantaneous water use efficiency *WUE*_i_ (**C**), and stomatal conductance *g*_s_ (**D**) of Ambrosia^TM^ apples on five rootstocks at the onset of heat stress in the second-year planting. Different letters in each sub-panel stand for significant differences amongst rootstocks (*P* ≤ 0.05, ANOVA, Tukey–Kramer pairwise comparisons; *n* = 6). The absence of letters stands for no significant differences.

At the onset of heat stress in August 2020, G.202 was the lowest in *P*_n_, electron transport rate (*ETR*) (Figure 7A), and variable fluorescence: maximum fluorescence ratio at steady-state photosynthesis lighting conditions (*Fv*’/*Fm*’) (Figure 7B). *WUE*_i_ was not significantly different amongst rootstocks (*P* ≤ 0.05), however G.202 showed slightly higher *WUE*_i_ with large tree-to-tree variations (Figure 7C). Leaf *P*_n_ (Figure 7A) and *g*_s_ (Figure 7D) were the highest in M.9 and B.9, followed by G.935 and M.26. Except for G.202, rootstocks showed higher *P*_n_ in the field than in the greenhouse (Figure 1), as a consequence of adapting to more abundant ambient photosynthetically active radiation (*PAR*).

**Figure 8:**
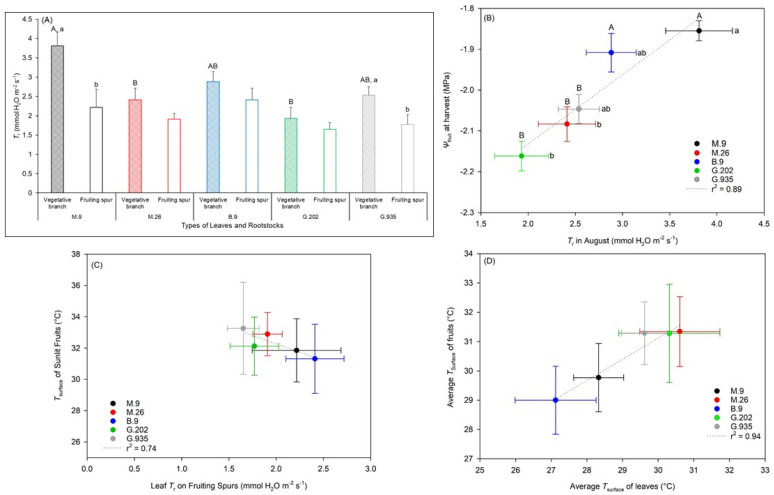
Leaf transpiration and its relationship to the tissue surface temperature of Ambrosia^TM^ apple on five rootstocks at the onset of heat stress in the second-year planting. (**A**) Transpirational rate *T*_r_ of leaves on vegetative branches and on fruiting spurs; (**B**) relationship between leaf *T*_r_ and water potential of fruit hypanthium *Ψ*_fruit_; (**C**) relationship between leaf *T*_r_ and surface temperature *T*_surface_ of sunlit fruits on fruiting spurs; and (**D**) relationship between average leaf *T*_surface_ and average fruit *T*_surface_. In (**A**), different uppercase letters stand for significant differences amongst rootstocks; different lowercase letters stand for significant differences between vegetative branches and fruiting spurs in each rootstock; in (**B**), different uppercase and lowercase letters stand for significant differences amongst rootstocks in *Ψ*_fruit_ and in leaf *T*_r_, respectively (*P* ≤ 0.05, ANOVA, Fisher LSD pairwise comparisons). The absence of letters stands for no significant difference.

Leaves on vegetative branches had higher *T*_r_ than those on fruiting spurs (Figure 8A). Leaf *T*_r_ on vegetative branches was the highest in M.9, followed by B.9 and G.935, and was the lowest in M.26 and G.202; higher leaf *T*_r_ corresponded to higher *Ψ*_fruit_ (Figure 8B, *r*^2^ = 0.89). On fruiting spurs, higher leaf *T*_r_ corresponded to a lower surface temperature (*T*_surface_) of adjacent sunlit fruits (Figure 8C, *r*^2^ = 0.74); however, rootstock difference was not significant. Average leaf *T*_surface_ was correlated with fruit *T*_surface_ (Figure 8D, *r*^2^ = 0.94); leaf *T*_surface_ was lower in B.9 than in M.26 (*P* = 0.04) and G.202 (*P* = 0.06).

## 3. Discussion

### 3.1. Rootstock Performances and Water Use Strategies

Semi-dwarfing and dwarfing rootstocks are commonly used to control scion vigor and alter yield efficiency in high-density plantings. Semi-dwarfing and dwarfing rootstocks usually produce apple trees that are about 60−90% and 30% to 60% of the size of seeded trees, respectively. Based on final trunk diameter and tree height, dwarfing rootstocks are further categorized into large, moderate, and small. In the field trial, the examined rootstocks rendered different scion trunk growth in Ambrosia^TM^ apple, as expected for their vigor classifications (Figure 5B). The semi-dwarfing G.202 had the largest *TCSA*, followed by the large dwarfing G.935, M.26 and M.9NIC29^®^. The small dwarfing B.9 produced the smallest *TCSA*. This trend was similar in a ten-year Honeycrisp rootstock trial in Summerland, where the tree vigor from high to low was G.202 > G.935 = M.26 > M.9 Pajam2 (similar vigor with M.9NIC29^®^) > B.9 [18,19]. 

#### 3.1.1. Semi-Dwarfing G.202

Larger rootstocks lead to lower yield efficiency, and vice versa. In the field trial, the semi-dwarfing G.202 led to the lowest yield efficiency, the lowest leaf chlorophyll concentration, and lower photosynthetic capacity (Figure 7A) and *g*_s_ (Figure 7D, Table 2). Firstly, it could indicate that as more resources were allocated to trunk growth, depleted nutrients might impair leaf function in chlorophyll synthesis and stomatal regulation. Secondly, lower leaf gas exchanges could suggest that its scion was exposed to water deficit, possibly caused by a hydraulic imbalance between the scion and the rootstock. Such imbalance often becomes more significant when the discrepancy between the scion demand and the rootstock supply aggravates. In addition, G.202 produced fruits with lower weight (Figure 6A), higher soluble solid content (Figure 6C), and lower *Ψ*_fruit_ (Figure 6E), which implied seasonal water deficit. Higher stomatal density was reported in the leaves of more vigorous rootstocks [20,21]; however, in the greenhouse experiment, G.202 led to the largest stomatal size but the lowest density in Ambrosia^TM^ scion leaves. This suggested that while higher stomatal density of rootstock leaves may be a good indicator for stronger vigor of rootstock itself, stomatal density of scion leaves may not show such correlation. Interestingly, the stomatal characteristics of G.202 were associated with the greatest decrease in *ΦCO*_2_ and *g*_s_, the greatest increase in *WUE*_i_, and the most severe defoliation by the end of the water deficit cycle (Table 1). Larger stomata might be prone to stomatal surplus under the medium level of photosynthetically active radiation (*PAR*) and in well-watered conditions, when the excessive increase in stomatal aperture and transpirational water loss were no longer coupled with photosynthetic gain. This could explain the concurrence of the decrease in *g*_s_ and the increase in *WUE*_i_ in G.202 when the soil water was depleted (Table 1). G.202 had similar *P*_n_ under *PAR* = 800 µmol photon m^−2^ s^−1^ in the greenhouse and under *PAR* = 1600 µmol photon m^−2^ s^−1^ in the field, suggesting that following defoliation, the remaining leaves photosynthesized at high capacity under relatively low *PAR* in the greenhouse. The efficiency of such adjustment is debatable, because maintaining older leaves as a photosynthate source during stress recovery may be a more valuable strategy rather than mobilizing nutrients into sink tissue during stress [22].

#### 3.1.2. Large Dwarfing G.935, M.26 and M.9

In the three large dwarfing rootstocks with intermediate trunk growth and yield efficiency, M.26 demonstrated the lowest leaf gas exchange rates (Figure 7A and Figure 8A) and the lowest *Ψ*_fruit_ in the field trial (Figure 6E). This was consistent with its low *P*_n_ and *T*_r_ under both well-watered and water deficit conditions, and its least soil water depletion by the end of dehydration cycle in the greenhouse experiment (Figure 1 and Figure 2, Table 1). It suggested that compared to M.9 and G.935, M.26 had lower water use, possibly attributed to its lower stomatal density and smaller stomatal area per leaf area (Figure 3A,C). By the end of the water deficit cycle, *ΦCO*_2_, *g*_s_ and *WUE*_i_ declined the most in M.26 amongst the three large dwarfing rootstocks (Table 1). There was also significant defoliation, suggesting severe water stress (Figure 4A). 

G.935 demonstrated modest water status and was slightly more vigorous than M.26 and M.9. Its fruits demonstrated several desirable quality attributes (Figure 6), such as higher and more consistent fruit weight and dry matter, and a higher sugar: acidity ratio—a parameter relevant to the sensory perception of apples [23,24]. Moderate scion growth and good fruit attributes suggested a well-balanced carbohydrate allocation between vegetative growth and fruit development. 

M.9 was a slightly less vigorous large dwarfing rootstock, although produced significantly larger trees than the small dwarfing B.9 (Figure 5B). Similar to B.9, the leaves on M.9 had stomata of a smaller size and higher density in the greenhouse (Figure 3). This could be attributed to the restricted rootstock water supply. Similarly, a study on the impact of deficit irrigation showed that water limitation led to higher stomatal density in Golden Delicious on EM-II rootstock [25]. In the field trial, leaf gas exchanges as well as the *T*_surface_ of leaves and fruits (Figure 7 and Figure 8) at the onset of heat suggested a better water status in M.9 than in the other large dwarfing rootstocks. Higher *Ψ*_fruit_, and moderate fruit size and dry matter (Figure 6), indicated that M.9 was likely exposed to less seasonal water stress. 

#### 3.1.3. Small Dwarfing B.9

The small dwarfing B.9 led to the slowest scion growth (Figure 5). During the greenhouse water-deficit cycle, it demonstrated the most stable leaf gas exchanges (Figure 1 and Figure 2) and the least defoliation (Figure 4), indicating more drought tolerance. This could be attributed to a lower hydraulic demand by a smaller scion accompanied with high stomatal density and moderate stomatal size (Figure 3). In the field trial, similar to M.9, B.9 showed high leaf gas exchanges as well as a low *T*_surface_ of leaves and fruits at the onset of heat stress (Figure 7 and Figure 8), and higher *Ψ*_fruit_ after storage, which suggested a good tree water status. Fruits on the two smallest rootstocks had higher firmness and a significantly lower sugar: acidity ratio, indicating the delayed maturation.

### 3.2. Responses to Water Deficit and Heat Stress in Relation to Water Use Strategies

#### 3.2.1. Drought Susceptibility versus Drought Resistance

Rootstocks influenced scion–water relationships, nutrient levels, and yield efficiency in Ambrosia^TM^ apple. This suggested that dwarfing intensities led to different water use strategies. Larger rootstocks, such as G.202, support more vigorous scion growth and higher yield potential, at a cost of more water use. This water use strategy is associated with wider scion xylem vessels and larger stomata. Such hydraulic characteristics may make the trees more susceptible to water deficit and heat stress. Under stresses, the overall leaf function declines, shown as chlorosis, reduction in *g_s_* and leaf gas exchanges, or the maintenance of *P*_n_ and chlorophyll concentration in limited leaves at the cost of defoliation, as observed in G.202 (Figure 1 and Figure 4). Therefore, good water management needs to be implemented to ensure tree health and fruit quality for such rootstocks. On the contrary, smaller rootstocks impose more hydraulic restriction on scions, leading to slower scion growth and lower yield potential; these rootstocks have less water demand, and consequently, can be more resistant to water deficit. 

#### 3.2.2. Drought Tolerance and Avoidance

The photosynthesis and transpiration of the studied rootstocks responded differently to soil water depletion and to the onset of heat stress, which indicated different mechanisms of stress resistance, i.e., tolerance and avoidance [26]. Drought tolerance is often associated with more resilient xylem transport and more flexible stomatal control; at the onset of stress, trees are able to maintain moderate and stable water use. M.9 and B.9 demonstrated such characteristics in this study. Higher stomatal density and smaller stomatal size enabled the leaves to fine-tune the stomatal regulation and to maintain *g*_s_ at a certain level for carbon assimilation and heat dissipation during stress (Figure 7 and Figure 8). This pinpointed the importance of understanding stomatal characteristics for predicting water use strategies and stress responses [27,28]. 

Drought-avoidant trees tend to avoid stress by decreasing water demand, via lowering *g*_s_ or reducing leaf area. This mechanism was observed in M.26 and G.935, shown as a reduction in leaf gas exchanges and *g*_s_, defoliation, lower stomatal area per leaf area in the water deficit experiment (Figure 1, Figure 2, Figure 3 and Figure 4), and relatively low photosynthesis, transpiration, *g*_s_ and *Ψ*_fruit_ in the field trial (Figure 6, Figure 7 and Figure 8). Due to the decrease in transpirational cooling, fruits on these rootstocks could have higher *T*_surface_ (Figure 8) and be exposed to more heat injury. The knowledge of different water use strategies and stress response mechanisms can facilitate the decision-making in rootstock selection and in irrigation management in accordance with the water use of specific rootstocks for stress alleviation and yield potential attainment. 

### 3.3. Stress Indicators for Ambrosia^TM^ Scion–Rootstock System

To reveal water use strategies and stress response mechanisms of different scion-rootstock systems, it often requires a comprehensive evaluation of tree–water relationships under a gradient of stresses, which involves the interpretation of multiple physiological parameters. In this study, stress indicators of leaves and fruits were explored. Their sensitivity to rootstocks and to the onset of stresses were assessed, based on their responsiveness and on their causal relationships (Table 3).

#### 3.3.1. Photosynthesis, Transpiration, and Leaf Surface Temperatures

Photosynthetic and transpirational capacities varied in rootstocks and in responses to stresses. Both leaf gas exchange rates and *g*_s_, and *ETR* and *Fv*’/*Fm*’, sensitively detected the differential rootstock responses to the onset of heat stress. Strong correlations existed amongst *P_n_*, *ETR*, *Fv*’/*Fm*’, *T*_r_ and *g*_s_. The Δ*ΦCO*_2_ was highly correlated with Δ*g*_s_ in the five examined rootstocks (Table 1; *r*^2^ = 0.98). This showed that the decrease in *g*_s_ under water deficit became a restricting factor for the quantum efficiency of CO_2_ assimilation in Ambrosia^TM^ apple leaves. These measurements can generate reliable information quickly and non-destructively, for early assessment on rootstock water use strategies: (1) large rootstocks that demand higher water use tend to demonstrate high leaf gas exchange rates under good water conditions, however their photosynthetic capacity and *g*_s_ rapidly decline to a much lower, symptomatic level under water deficit, which suggests drought susceptibility. Such susceptibility can expose the trees to more subsequent heat stress, due to the compromised transpirational cooling effect, which is often observed as a concurrence of decreased *T*_r_ and increased average tissue *T*_surface_; (2) drought-avoidant scion–rootstock systems typically reduce *g*_s_ at the onset of water deficit, to conserve water and to continue leaf gas exchanges at a lower level; (3) drought-tolerant rootstocks support less vigor and demand less water. They are capable of maintaining leaf gas exchanges at a moderate and steady level; their relatively high *ETR* and *Fv*’/*Fm*’ indicate good water status at the onset of presumed stresses. Other traits of tolerance include the lower *T*_surface_ of leaves and fruits, higher *Ψ*_fruit_, and less extent of leaf chlorosis and defoliation. Lower scion hydraulic conductance and elevated abscisic acid may be involved in the better adaptation of smaller scions to water-limited environments [29]. 

Calculated based on instantaneous carbon gain *P*_n_ against water loss *T*_r_, *WUE*_i_ should be interpreted carefully with reference to other parameters such as *P*_n_, *g*_s_, and longer-term *WUE* [30]. Increased *WUE* can be a consequence of either enhanced photosynthetic carbon gain, or reduced water loss caused by various factors, and therefore, it does not necessarily correspond to higher yield potential. Increased *WUE* at the cost of significantly reduced water use can be associated with decreased yield [31], as in drought-susceptible and drought-avoidant crops under water-limited conditions. On the contrary, scion–rootstock systems with drought tolerance tend to maximize soil moisture use; higher *g*_s_ and lower *WUE* may associate with higher carbon gain and higher yield potential, given that soil moisture stays away from extreme deficits. 

Leaf *T*_r_ on vegetative branches was higher than that of on fruiting spurs, likely due to less water availability in fruiting spur leaves due to fruit–leaf competition for water. The leaves on vegetative branches were more responsive to the onset of heat stress (Figure 8A); average *T*_surface_ showed more variation amongst rootstocks than *T*_surface_ of directly sunlit tissues did. This suggested that the average *T*_surface_ of leaves on vegetative branches could be a more accurate indicator for the tree water status. However, it showed less sensitivity and more leaf-to-leaf variation than leaf gas exchanges, *ETR* and *Fv*’/*Fm*’, which made it a less effective stress indicator. A similar insensitivity of leaf temperature was also reported in a plant–water relationship study on young peach trees [32]. 

#### 3.3.2. Water Potential and Quality Attributes of Fruits

Mid-day stem water potential has been widely used in plant water status assessment since the invention of the hydrostatic pressure method [33]. In fruit trees, it is well correlated with leaf transpiration [34]. However, the data collection involves destructive sampling and subjective judgement by the operator of the pressure chamber. The optimal time window for measurement is narrow, due to its strong diurnal pattern under the impact of leaf transpirational water loss. In comparison, *Ψ*_fruit_ varies less diurnally because of the relatively low surface conductance and transpirational rate in fruits. Its main component, the osmotic potential, is determined by the number of soluble solids and water availability in fruits. Previous studies showed it was an important parameter in seasonal fruit water relations, which could demonstrate the effects of irrigation and crop load on fruit and tree water status [35,36]. In this study, *Ψ*_fruit_ was negatively correlated with fruit *SSC*, and positively correlated with leaf *T*_r_ (Figure 8B), which suggested that it could be an effective water status indicator. Fruit weight, size, and dry matter accumulation are largely determined by fruit expansion and carbohydrate allocation, both impacted by water availability [37]. In turn, the variations in fruit weight and dry matter can reflect the changes in seasonal water availability. The concurrence of lower fruit weight and higher *DM*% or *SSC*% (i.e., less water content %), often indicates the reduction in fruit expansion and carbohydrate accumulation caused by seasonal water deficit (Table 3).

### 3.4. Summary and Future Perspectives

Ambrosia^TM^ apples on semi-dwarfing, large dwarfing, and small dwarfing rootstocks performed differentially under greenhouse and field conditions, also responding differentially to water deficit and heat stress. Plant–water relationships suggested different water use and stress response strategies in the rootstocks of different vigor levels. The semi-dwarfing G.202 led to the largest scion growth and demonstrated more water demand and more susceptibility to stresses. Large dwarfing rootstock G.935 and M.26 showed more stringent stomatal control and reduced water use at the onset of stresses, to avoid water loss. The smallest large dwarfing M.9 and the small dwarfing B.9 appeared to be more drought tolerant, and maintained leaf functions at a moderate but stable level. Future studies should be extended to other apple cultivars, to investigate whether these rootstocks would influence scion–water relationships in a way similar to Ambrosia^TM^. More knowledge about scion–rootstock water use strategies can facilitate the decision-making on irrigation management, to alleviate stresses and achieve production goals for specific rootstock vigor categories. 

In the Ambrosia–rootstock systems, the sensitivity of stress indicators from high to low was leaf gas exchanges ≈ *ETR* ≈ *Fv*’/*Fm*’ > leaf *T*_surface_ > chlorosis and defoliation in leaves, and *Ψ*_fruit_ ≈ *SSC*% > fruit *T*_surface_ > fruit weight in fruits (Figure 6, Figure 7 and Figure 8, Table 3). Strong correlations existed between leaf photosynthetic parameters, leaf transpirational rate, and *Ψ*_fruit_ (Figure 7 and Figure 8).

This study set the first small step towards a comprehensive understanding of water relationships in Ambrosia^TM^ scion–rootstock systems, and of the sensitivity and relationship of multiple stress indicators. In future studies, developmental traits such as stomatal size and density, leaf area, and trunk increment should be re-evaluated under varied field conditions and at different tree ages; controlled field experiments should be conducted under aggravating effects of water deficit, heat stress, and heavy crop load. Multifaceted physiological studies on root–shoot–fruit dry matter partitioning, shoot-grafted union–root hydraulic conductivity, as well as scion–rootstock differentiations in xylem vessels and symplastic water transport, will provide more conclusive evidence for the speculated rootstock-specific water use and stress response strategies. 

## 4. Materials and Methods

### 4.1. Plant Materials and Growth Conditions

#### 4.1.1. Greenhouse Conditions of Water Deficit Experiment

Two-year-old Ambrosia^TM^ trees grafted on five rootstocks, i.e., M.9, M.26, B.9, G.202 and G.935 (*n* = 10 trees for each rootstock, in randomized locations in the greenhouse compartment), were planted in SunShine^®^ Mix # 4 professional growing mix (Sungro, Agawam, MA, USA), in 20 gallon nursery pots (height: 55 cm; diameter: 44.5 cm), during October 2018–February 2019. Supplementary light was provided for 16 h per day. Air temperature was 22–24 °C in daytime and 16–18 °C at night. Relative humidity was 50–60%. Trees were irrigated to full water holding capacity every three days, and the fertilizer 20-20-20 was applied monthly at a rate of 1 g/1 L. The water deficit experiment was conducted in January 2019. During the dehydration cycle, trees were not irrigated for one week to allow soil water depletion, and then the pots were recovered to full water-holding capacity. Volumetric water content (*VWC*) was monitored in the topsoil of 5 cm depth using 5TM soil sensors and was recorded at 30 min interval using an EM50 data logger (Meter Environment, Pullman, WA, USA). The variation in *VWC* in the beginning and at the end of the dehydration cycle (Δ *VWC*_Day 7–Day 1_, m^3^/m^3^) was analyzed to demonstrate the change in soil moisture in the pots of each rootstock (*n* = 3 pots).

#### 4.1.2. Field Trial Conditions 

Three-year-old Ambrosia^TM^ trees grafted on M.9, M.26, B.9, G.202 and G.935 were grown in silt–loam soil at the experiment farm (49°33′45″ N, 119°38′55″ W, elevation 454 m) in complete randomized block design (*n* = 6 plots for each rootstock, 3 trees per plot). Under the typical South Okanagan climate, the growing season starts with a cool and moist spring, followed by a hot and dry summer, with daily maximum temperature of 28.4 °C in July and 28 °C in August (Environment Canada), and historical average moisture deficit of 157 mm in July and 133 mm in August (data acquired from www.farmwest.com, BC-Okanagan South-Summerland EC; Access date: 27 December 2020). In 2020, precipitation and average moisture deficit were 15 mm and 155 mm in July, and 11 mm and 140 mm in August, respectively. Irrigation was applied through drip lines from 08:00 to 10:00 a.m. every three days from May to early October. Trees were trained to Tall Spindle Axe structures in high-density planting. Blossoms were hand-thinned in May 2020, followed by fruitlet thinning in June, to keep five fruits per tree. Stress responses were evaluated under low *VWC* and high air temperature in August. Yield was assessed at harvest in late September. 

### 4.2. Measurements of Stress Indicators of Plant–Water Relationships

#### 4.2.1. Leaf Photosynthesis and Transpiration

Leaf photosynthetic rate (*P*_n_), transpirational rate (*T*_r_) and stomatal conductance (*g*_s_) were measured using the infrared gas analyzer of LICOR-6800 Portable Photosynthesis System (LICOR, Lincoln, NE, USA). Instantaneous water use efficiency (*WUE*_i_) [30] was calculated as *P*_n_ divided by *T*_r_, both parameters being measured simultaneously at a stable photosynthetically active radiation level (*PAR*). Relative electron transport rate (*ETR*), maximum quantum efficiency of CO_2_ assimilation (*ΦCO_2_*), and the variable fluorescence: maximum fluorescence ratio at steady-state photosynthesis lighting conditions (*Fv*’/*Fm*’), were measured using the chlorophyll fluorometer of LICOR-6800, to represent *Y*(II), i.e., the relative quantum efficiency of Photosystem II (PSII) [38]. To simulate the ambient mid-day radiation, *PAR* in the LICOR-6800 sample chamber was set at 800 µmol photon m^−2^ s^−1^ in the greenhouse experiment, and at 1600 µmol photon m^−2^ s^−1^ in the field trial. Other environmental parameters were set as flow rates at 700 µmol s^−1^, chamber relative humidity at 50%, and the CO_2_ concentration of sample chamber at 400 µmol CO_2_ m^−2^ s^−1^. Temperature in the sample chamber was set as equal to the ambient. In the greenhouse experiment, leaf gas exchange measurements were conducted at 24 h (Day 1), 96 h (Day 4) and 168 h (Day 7) after irrigation (dehydration cycle), and then at 24 h after irrigation was resumed (rehydration). In the field trial, leaf gas exchanges and *Y*(II) parameters were measured 72 h after irrigation on 2 days in August when *VWC* declined below 0.15 m^3^/m^3^ and the maximum air temperature was 37.1–38.3 °C. The measurements were scheduled between 08:00 a.m. and 01:00 p.m., with the sequence of measurements randomized amongst rootstocks (*n* = 6).

#### 4.2.2. Tissue Surface Temperature and Fruit Water Potential, *Ψ*_fruit_

In the field trial, thermal images of sunlit apple fruits, and of sunlit and shaded leaves, were captured using FLIR E8 Infrared camera (FLIR^®^ Systems Inc., Wilsonville, OR, USA; 7 mm focal lens, 320 × 240 IR resolution), immediately before the photosynthesis measurements were conducted on the adjacent leaves. Surface temperature (*T*_surface_) of fruits and leaves was automatically identified on the thermal images by the software FLIR Tools (V. 6.4, FLIR^®^ Systems Inc.) (*n* = 6). Average fruit *T*_surface_ was calculated as the mean temperature of sunlit skin and shaded skin of the fruit. Average leaf *T*_surface_ was calculated as the mean temperature of the sunlit and shaded leaves. 

Tissue water potential of fruit hypanthium (*Ψ*_fruit_) was measured using a WP4C potentiameter (Meter Environment). Fruits were collected at optimal harvest time, stored in air at 4 °C for four weeks, and recovered to 23–24 °C in laboratory conditions prior to measurement (*n* = 24 fruits per rootstock). The instrument was calibrated using −2.19 MPa standard KCl solution. One slice of fruit hypanthium tissue of 3–4 mm thick was cut out vertically from the fruit, and then a stainless sample cup was used to excise one disc of sample from the centre of the hypanthium slice, with the rim of sample cup at about 1 cm apart from the fruit skin. The disc of sample was gently pushed down to the bottom of the sample cup to half-fill the cup space. Fruit tissue residual was quickly and carefully wiped from the rim and the outside of the cup. When possible, the sample was placed in WP4C immediately after being excised. When a waiting time was inevitable, the cup lid was capped tightly to restrict water transfer until the instrument was ready to load sample. Measurements were automatically completed after 6–10 min equilibration in the fast mode.

### 4.3. Leaf Chlorophyll Concentration and Stomatal Characteristics

Leaf chlorophyll concentration was assessed in absolute units of µmol of chlorophyll per m^2^ of leaf area, using the apple setting in the MC-100 Chlorophyll Meter (Apogee Instruments Inc., Logan, UT, USA) [39], on the same days of photosynthesis measurements. Five healthy, fully developed, and sunlit leaves per tree were randomly sampled non-destructively, to represent the average leaf chlorophyll concentration for each tree (*n* = 6). By the end of the greenhouse water deficit experiment, leaf area of defoliated leaves per tree was estimated by multiplying the dry weight of fallen leaves with 264.8 cm^2^/g, i.e., the coefficient of leaf area: leaf dry weight for Ambrosia^TM^ apples. 

For stomatal density and size analysis, a leaf imprint was made by painting a 5 mm strip of transparent nail polish on the interveinal area on the abaxial side of leaves. The completely dried nail polish film was peeled off and mounted in a drop of water on the microscopic slide. Five imprints were prepared per leaf, and 4 leaves were sampled from each of 6 trees per rootstock. The leaf imprints were observed in the mode of transmitted differential interference contrast under the 10× objective lens of Zeiss Axio microscope (Zeiss, Oberkochen, Germany). Images were acquired using AxioCam MR R3 camera (Zeiss) and software Zen Pro 2012 (Zeiss). The number of stomata per mm^2^ of leaf area was counted in the software ImageJ (National Institutes of Health, V.1.53) [40] to represent stomatal density. The lengths and widths of leaf stomata were measured on 10 random stomata per imprint using the same software. The size of the stomata was estimated as the area of the ellipse, i.e., π × 1/2 length × 1/2 width. Stomatal area per leaf area was calculated by multiplying stomatal density with the average size of stomata per unit of leaf area. 

### 4.4. Nutrient Use and Yield Efficiency

Trunk cross-sectional diameter was measured at 30 cm above the grafted union in north–south direction and east–west direction, using a Digimatic 8 ABS digital caliper with DP-1VA Digimatic data logger (Mitutoyo America Corporation, Renton, WA, USA). Trunk cross-sectional area (*TCSA*) was calculated in an approximation of a round disc, to represent the vigor of scion growth (*n* = 10 trees per rootstock in the greenhouse experiment; *n* = 6 plots per rootstock in the field trial, with 3 trees per plot). In the field trial, yield efficiency was calculated as the kilograms of fruits at harvest per cm^2^ of *TCSA*. Fruits without any disorders were sampled at harvest (*n* = 3; each sample consisted of 2 slices per fruit, 3 fruits per tree, 2 trees per rootstock), completely dehydrated at 80 °C in an oven for one week, and sent to A&L Canada Laboratories Inc. (London, ON, Canada) for the complete mineral nutrient analysis. 

### 4.5. Fruit Quality Attributes

At harvest, fresh weight and dry matter percentage (*DM*%) of individual fruits were measured using a compact bench scale (Ohaus R71MHD35 Ranger 7000, Parsippany, NJ, USA) and Felix-750 Produce Quality Meter (Felix Instruments Inc., Camas, WA, USA), respectively (*n* = 6, 5 fruits for each replication). Fruit fresh weight was multiplied by *DM*% to estimate dry matter weight per fruit in grams (fruit dry matter weight).

Soluble solid content (*SSC*, in °Brix%) and titratable acidity (*TA*, in malic acid g/100 mL) were evaluated after four weeks of air storage at 4 °C. Two slices from each of 6 apples were combined and juiced to prepare one juice sample for *SSC* and *TA* (*n* = 6 juice samples per rootstock). *SSC* was measured using a portable refractometer (30P, Mettler Toledo, Columbus, OH, USA). *TA* was assessed by end-point titration, using OrionStar T940 titrator (Thermo Scientific, Waltham, MA, USA). Fruit firmness was measured using Fruit Texture Analyzer (FTA-G25, GÜSS Manufacturing Ltd, Strand, South Africa) (24 fruits per rootstock).

### 4.6. Statistical Analysis

Significant differences were analyzed by ANOVA, Fisher LSD, or Tukey–Kramer pairwise comparisons (*P* ≤ 0.05), using OriginPro 8.0 (OriginLab, Northampton, MA, USA). The number of replications is described in each method above. Data plotting and linear regressions were conducted in Sigma Plot (V.13.0, Systat Software Inc., San Jose, CA, USA). Box plots showed the minimum, first quartile, median, third quartile, and maximum values. Bars or round discs with error bars were means ± standard errors. Linear regression was shown as dot lines; *r*^2^ referred to the coefficient of determination for correlation. Different letters in each sub-panel indicated significant differences; the absence of letters indicated no significant difference. 

## Figures and Tables

**Table 1:** Changes in instantaneous water use efficiency (*WUE*_i_), stomatal conductance (*g*_s_) and maximum quantum efficiency of CO_2_ assimilation (*ΦCO*_2_) of Ambrosia^TM^ scion on five rootstocks, along with the decline in soil volumetric water content (*VWC*) from Day 1 to Day 7 after irrigation in the greenhouse experiment.

Rootstocks	Δ*VWC*_Day 7–Day 1_ (m^3^/m^3^)	Δ*WUE*_i Day 7–Day 1_ (µmol CO_2_/mmol H_2_O)	Δ*g*_s Day 7–Day 1_ (mmol m^−2^ s^−1^)	Δ*ΦCO*_2 Day 7–Day 1_ (mmol CO_2_ mol^−1^ Absorbed Quanta)
M.9	−0.31 ± 0.03 a	0.16 ± 0.19 b	−9.14 ± 3.61 b	−1.04 ± 0.37 b
M.26	−0.14 ± 0.02 b	−0.83 ± 0.40 c	−21.72 ± 3.84 b	−2.59 ± 0.60 ab
B.9	−0.25 ± 0.01 ab	−0.39 ±0.26 bc	7.32 ± 12.31 b	−0.34 ± 1.26 b
G.202	−0.25 ± 0.06 ab	1.14 ± 0.34 a	−67.46 ± 12.30 a	−5.37 ± 1.82 a
G.935	−0.3 ± 0.01 a	0.21 ±0.21 b	−16.46 ± 12.40 b	−1.68 ± 0.72 b

Note: Data of Δ are the values in Day 7 subtracted by the values in Day 1, shown as the mean ± standard error. Different letters in each column indicate significant differences amongst rootstocks (*P* ≤ 0.05, ANOVA, Fisher LSD pairwise comparisons) (*n* = 3 for Δ *VWC*_Day 7–Day 1_; *n* = 6 for Δ*WUE*_i_, Δ*g*_s_ and Δ*ΦCO*_2_).

**Table 2:** Leaf chlorophyll concentration, fruit mineral nutrient levels and yield efficiency of Ambrosia^TM^ scions on five rootstocks in the second-year planting in 2020.

		Rootstocks				
		M.9	M.26	B.9	G.202	G.935
Leaf Chlorophyll (µmol m^−2^)		452.7 ± 2.7 a	441.2 ± 2.7 a	450.7 ± 1.5 a	398.4 ± 9.1 b	448.6 ± 1.7 a
Fruit Nutrients	N	7.91 ± 0.42 a	5.54 ± 0.27 b	6.00 ± 0.14 b	4.96 ± 0.13 b	9.13 ± 0.98 a
	P	2.00 ± 0.11 bc	2.11 ± 0.04 b	1.78 ± 0.08 c	2.42 ± 0.05 a	2.61 ± 0.06 a
	K	25.66 ± 0.68 a	24.54 ± 0.61 a	22.62 ± 0.23 ab	22.85 ± 2.41 ab	18.36 ± 2.37 b
	Mg	1.37 ± 0.05 a	1.47 ± 0.07 a	1.24 ± 0.11 ab	1.18 ± 0.09 ab	1.06 ± 0.11 b
	Ca	0.84 ± 0.05 ab	1.04 ± 0.03 a	1.11 ± 0.04 a	0.58 ± 0.04 bc	0.31 ± 0.17 c
	B	0.032 ± 0.001 c	0.033 c	0.028 c	0.051 ± 0.001 b	0.064 ± 0.007 a
	Zn	0.003	0.004	0.005 ± 0.001	0.004	0.004
Yield Efficiency (kg/cm^2^ *TCSA*)		0.44 ± 0.04 b	0.38 ± 0.02 bc	0.57 ± 0.03 a	0.32 ± 0.03 c	0.36 ± 0.02 bc

Note: Fruit nutrients are in the unit of mg/100 g fresh weight. Data are shown as the mean ± standard error. Different letters in each row indicate significant differences amongst rootstocks (*P* ≤ 0.05, ANOVA, Fisher LSD pairwise comparisons), whereas the absence of letters stands for no significant difference.

**Table 3:** Growth characteristics and stress indicators of Ambrosia^TM^ scion–rootstock systems with different vigor and water use strategies.

Water Use Strategy	Growth Characteristics			Responses of Physiological Indicators to Stresses					
	*TCSA*	Yield Potential	Yield Efficiency	PSII Efficiency (*ETR*, *Fv*’/*Fm*’)	*P*_n_, *ΦCO*_2_, *T*_r_, *g*_s_	Average *T*_surface_ of Leaves and Fruits	*Ψ*_fruit_	Fruit Weight, *DM*%, *SSC*%	Leaf Chlorosis, Defoliation
High Demand, Drought Susceptibility	Larger	High	Low	Low	Symptomatic reduction	More elevated	Low	Lower weight, higher *DM*% and *SSC*%	Severe
Reduction in Water Use, Drought Avoidance	Medium	Medium	Medium	Medium–normal	Rapid reduction to a lower level	More elevated	Low	Higher *DM*% and *SSC*%	Moderate defoliation
Less Water Use, Drought Tolerance	Smaller	Medium	High	Normal	Moderate and stable	Less elevated	High	Lower *DM*% and *SSC*%	Slight–none

## Data Availability

Raw data used to generate figures and tables are available on request.
